# Outcomes of Comprehensive Care for Children Empirically Treated for Multidrug-Resistant Tuberculosis in a Setting of High HIV Prevalence

**DOI:** 10.1371/journal.pone.0037114

**Published:** 2012-05-22

**Authors:** Hind Satti, Megan M. McLaughlin, David B. Omotayo, Salmaan Keshavjee, Mercedes C. Becerra, Joia S. Mukherjee, Kwonjune J. Seung

**Affiliations:** 1 Partners In Health, Maseru, Lesotho; 2 Department of Global Health and Social Medicine, Harvard Medical School, Boston, Massachusetts, United States of America; 3 Division of Global Health Equity, Brigham and Women’s Hospital, Boston, Massachusetts, United States of America; University of Witwatersrand, South Africa

## Abstract

**Background:**

Few studies have examined outcomes for children treated for multidrug-resistant tuberculosis (MDR-TB), including those receiving concomitant treatment for MDR-TB and HIV co-infection. In Lesotho, where the adult HIV seroprevalence is estimated to be 24%, we sought to measure outcomes and adverse events in a cohort of children treated for MDR-TB using a community-based treatment delivery model.

**Methods:**

We reviewed retrospectively the clinical charts of children ≤15 years of age treated for culture-confirmed or suspected MDR-TB between July 2007 and January 2011.

**Results:**

Nineteen children, ages two to 15, received treatment. At baseline, 74% of patients were co-infected with HIV, 63% were malnourished, 84% had severe radiographic findings, and 21% had extrapulmonary disease. Five (26%) children had culture-confirmed MDR-TB, ten (53%) did not have culture results available, and four (21%) subsequently had results indicating drug-susceptible TB. All children with HIV co-infection who were not already on antiretroviral therapy (ART) were initiated on ART a median of two weeks after the start of the MDR-TB regimen. Among the 17 patients with final outcomes, 15 (88%) patients were cured or completed treatment, two (12%) patients died, and none defaulted or were lost to follow-up. The majority of patients (95%) experienced adverse events; only two required permanent discontinuation of the offending agent, and only one required suspension of MDR-TB treatment for more than one week.

**Conclusions:**

Pediatric MDR-TB and MDR-TB/HIV co-infection can be successfully treated using a combination of social support, close monitoring by community health workers and clinicians, and inpatient care when needed. In this cohort, adverse events were well tolerated and treatment outcomes were comparable to those reported in children with drug-susceptible TB and no HIV infection.

## Introduction

Between 2000 and 2009, there have been an estimated five million new cases of multidrug-resistant tuberculosis (MDR-TB), defined as resistance to isoniazid and rifampicin. 3.5 million patients received no treatment or treatment of unknown quality, and 1.5 million died [1]. An estimated 10% to 15% of these patients were children [2]. Pediatric MDR-TB is often a result of primary resistance transmitted from adults with MDR-TB, rather than secondary resistance acquired as a result of suboptimal therapy [3], [4]. Thus, unlike adult MDR-TB prevalence, which often reflects the failure of national TB control programs, pediatric MDR-TB surveillance reveals ongoing transmission of MDR-TB in the community.

Pediatric tuberculosis presents several unique diagnostic and treatment challenges. Treatment delay is particularly problematic for children with MDR-TB, and outcomes are poor when treatment is delayed [5], [6]. Because of the difficulty of obtaining sputum from young children [7] and their tendency to have paucibacillary TB [6], [7], children are less likely to have culture confirmation of TB [8]–[10]. Thus, drug-susceptibility testing (DST) is often not possible [5], and the decision to initiate second-line TB therapy in children with clinical and radiographic evidence of TB disease must be made based on the patients’ TB history [11]–[13].

Additional challenges arise when diagnosing and managing MDR-TB in the context of HIV co-infection. Poor response to first-line TB treatment in HIV co-infected patients can occur for reasons other than drug resistance, including poor adherence and the presence of other opportunistic infections with pulmonary manifestations, and HIV co-infected patients are at increased risk of recurrent TB infections [14], [15]. Moreover, patients failing first-line TB treatment are often hospitalized in facilities lacking in infection control, putting the patient at risk of being re-infected with circulating drug-resistant TB strains or transmitting TB to others. Outbreaks of MDR-TB among HIV patients in health facilities have been well-documented [16]–[21], including a nosocomial outbreak in a pediatric ward [22]. Additionally, optimal timing of antiretroviral therapy (ART) initiation relative to second-line TB therapy is unknown [23]. Patients on treatment for both HIV and MDR-TB face a high pill burden, and little is known about the frequency and severity of adverse events from concomitant treatment, particularly in children [22]–[24].

A small number of studies and case series have examined outcomes for children treated for MDR-TB [6], [25]–[32]. Taken together, these suggest that when appropriate treatment–including individualized regimens and strong supervision and support–is initiated early, favorable treatment outcomes can be achieved in pediatric MDR-TB patients. However, there are few reports of DR-TB/HIV co-treatment in pediatric patients [22], [30], [31].

We sought to describe the clinical features, treatment course, and final outcomes in a cohort of children treated for MDR-TB in Lesotho using a community-based treatment delivery model.

## Methods

### Ethics Statement

As this was a retrospective study of medical information previously collected in the course of routine clinical care, informed consent was not required. This study was approved by the Partners HealthCare Human Research Committee.

### Setting

Lesotho is a small, mountainous country completely surrounded by South Africa, with a population of approximately 1.8 million. Lesotho has a high burden of both HIV and TB: 24% of the adult population is HIV infected [33], and the estimated prevalence of TB is 402 cases per 100,000 population [34]. Among TB patients, an estimated 77% are co-infected with HIV [34]. Since 2007, the Lesotho Ministry of Health and Social Welfare (MOHSW), with support from Partners In Health, has operated a national MDR-TB program offering comprehensive, community-based treatment, free of charge to patients and their families. Partners In Health is a non-governmental organization that has worked in partnership with local communities and governments to provide health care access to a combined population of 2.4 million people in 12 countries, including more than 13,000 MDR-TB patients in Peru, Russia, Kazakhstan, and Lesotho [35].

### Study Design

We conducted a retrospective analysis of medical charts of all patients ages 15 years or younger who initiated second-line TB treatment for confirmed or suspected MDR-TB between July 27, 2007 and January 1, 2011. We excluded one patient who began an MDR-TB regimen and was subsequently switched to first-line tuberculosis treatment based on drug-susceptibility testing (DST) results, patient history, and clinical and radiographic response to MDR-TB treatment.

### Treatment Strategy

Treatment protocols, including pediatric dosing, followed international standards of care [25], [36]–[38]. In general, patients were referred by clinicians working in the MOHSW network of hospitals and health centers to the Lesotho national MDR-TB program for evaluation of confirmed or suspected MDR-TB. At the time of initial evaluation, sputum or lymph node specimens were collected and sent for DST. While awaiting DST results, a child was initiated on an empiric regimen with second-line TB drugs if he or she met the following criteria: 1) the child had clinical and radiographic evidence of TB disease, and 2) the child was either a household contact of a patient with confirmed or presumed MDR-TB, or the child had failed at least one supervised first-line regimen. The protocol for initiating empiric treatment has been described in detail previously [39]. Empiric regimens consisted of six drugs–pyrazinamide, capreomycin (or kanamycin), levofloxacin (or moxifloxacin or ofloxacin), ethionamide (or prothionamide), cycloserine, and para-aminosalicylic acid (PAS). When DST results of the patient or household source case were available or when a patient had received second-line drugs previously, treatment regimens were modified accordingly.

The management of patients who were started empirically on MDR-TB treatment and subsequently had DST results indicating susceptibility to isoniazid and rifampicin was considered on an individual case-by-case basis. Based on the patient’s clinical history, duration of MDR-TB treatment, and treatment response, the clinical team decided whether to transfer the patient to the local health center to receive first-line TB treatment or maintain the patient on the second-line regimen with the addition of rifampicin and high-dose isoniazid.

Lesotho experienced an uninterrupted supply of second-line antituberculous drugs during the study period. Because none of antituberculous drugs used in the standard regimen were available in pediatric formulations, patients were provided with adult formulations, and the clinicians and community health workers instructed caregivers to divide doses and cut tablets into the appropriate size.

Patients with unknown HIV serostatus were offered testing at the first visit. All patients with HIV co-infection who were not yet receiving ART were initiated on an ART regimen as soon as they were tolerating the MDR-TB treatment regimen, regardless of CD4 cell count. The standard ART regimen consisted of two nucleoside reverse transcriptase inhibitors (zidovudine or stavudine plus lamivudine) and one non-nucleoside reverse transcriptase inhibitor (efavirenz, or nevirapine for children under three years of age). All patients received pyridoxine supplementation to prevent peripheral neuropathy [40].

MDR-TB treatment was provided in the community for patients in stable, ambulatory condition. Trained, paid community health workers (CHWs) delivered second-line antituberculous drugs and antiretroviral therapy twice-daily under direct observation. The CHWs also monitored patients daily for adverse events. Routine laboratory monitoring–including full blood count, liver function tests, electrolytes, and creatinine–was performed monthly at clinic visits, and a specialist clinician evaluated patients at these visits. Thyroid-stimulating hormone (TSH) was initially measured within the first six months of MDR-TB treatment initiation and subsequently every four to six months. Patients with at least one TSH result elevated above 10.0 mIU/l were initiated on a weight-based dose of levothyroxine, which was continued until one month after completion of MDR-TB therapy; TSH was monitored throughout the treatment period at regular intervals [41]. Patients with a serum potassium value below 3.5 mmol/l received potassium and magnesium supplementation until normalization of serum potassium. Patients experiencing nausea or vomiting were treated with prochlorperazine. Chest radiographs were taken at baseline, every six months, and at treatment completion, and the clinical team attempted to obtain sputum samples for culture of *M. tuberculosis* at baseline and at each monthly clinic visit.

A community team communicated regularly between the clinics and the community, tracking patients who missed any appointment and assessing the stability of each patient’s household situation. The community team provided patients and their caregivers with counseling about MDR-TB and HIV, food supplements, reimbursement for treatment-related transportation, and other types of psychosocial support as needed. Patients who were clinically unstable or experienced severe adverse events were admitted to Botsabelo Hospital, a specialized MDR-TB inpatient facility in the capital (Maseru).

### Data Collection and Analysis

Baseline characteristics, treatment history, and clinical signs and symptoms, including radiographic findings, laboratory values, adverse events, and treatment outcomes, were abstracted from patient records and entered into a Microsoft Access 2003 (Microsoft Corporation, Redmond, WA) database. Standard definitions for retrospective MDR-TB analyses were used to determine final treatment outcomes [42]. Cure was defined as five consecutive negative cultures at least 30 days apart in the final 12 months of treatment, and treatment failure was defined as two or more positive cultures in the final year of treatment or a positive result for any of the final three cultures. Patients were designated treatment completed if they completed treatment according to national protocol but did not meet the definition of cure or treatment failure. Data were analyzed using SAS 9.2 (SAS Institute, Cary, NC).

## Results

**Figure 1:**
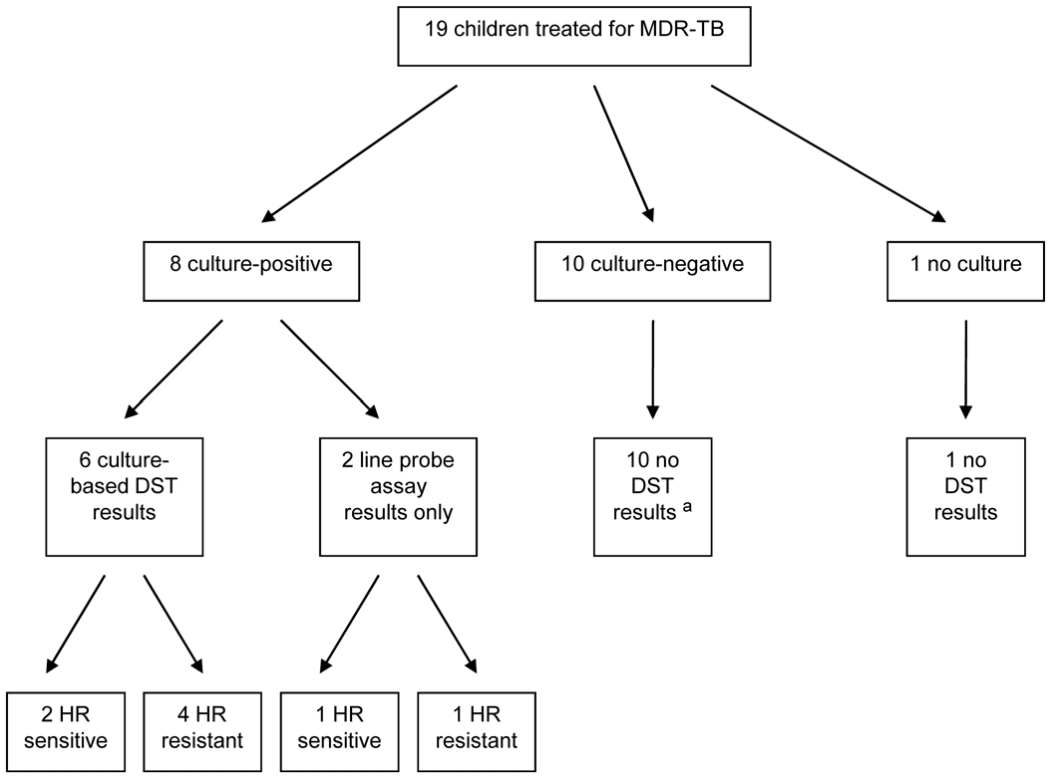
Baseline bacteriologic results for pediatric patients treated for MDR-TB. Note: H: isoniazid; R: rifampicin; DST: drug-susceptibility testing. ^a^ One patient (case 19) was culture-negative at baseline but later had DST results, indicating susceptibility to all drugs tested, from a positive culture in the tenth month of treatment.

Nineteen children were included in this analysis (Table 1). At the start of treatment, their ages ranged from two to 15 years old, with a median age of eight years (Table 2). The cohort was characterized by a high prevalence of baseline malnutrition (63%), stunting (63%), HIV co-infection (74%), and severe radiographic findings (cavitary lesions or bilateral disease) (84%). Fifty-three percent of the patients had received two or more previous courses of TB treatment prior to referral. All patients presented with pulmonary manifestations of TB infection, and four patients presented with concurrent extrapulmonary TB (three cases of miliary TB and one case of tuberculous lymphadenitis). Ten patients (53%) were smear-negative at the time of MDR-TB treatment initiation. Figure 1 shows the baseline culture and DST results of the 19 patients.

**Table 1:** Baseline clinical and demographic characteristics of the first 19 children treated for MDR-TB in Lesotho, 2007–2011.

Case	Age (years)	Clinical presentation	Smear	Culture	Chest radiography	Household source case	Number of previous courses of TB treatment	Drugs received previously
1	13	PTB	Pos	Pos	Bilateral infiltrates, collapsed left lung	TB patient who died on treatment	4	H, R, Z, E, Eto, Cfx
2	3	PTB and EPTB (miliary)	Neg	Neg	Bilateral miliary pattern	Confirmed MDR-TB patient	1	H, R, Z, E
3	8	PTB and EPTB (miliary)	Neg	Neg	Bilateral miliary pattern	TB patients who died on treatment	2	H, R, Z, E, Cfx
4	8	PTB	Pos a	Pos a	Bilateral infiltrates, unilateral cavity	TB patients who died on treatment	1	H, R, Z
5	13	PTB	Pos	Pos	Unilateral infiltrates	None	1	H, R, Z, E
6	9	PTB	Neg	Neg	Bilateral infiltrates	None	1	H, R, Z, E
7	15	PTB	Pos	Pos	Bilateral infiltrates, bilateral fibrosis, unilateral cavities	TB patients who died on treatment	2	H, R, Z, E, S
8	5	PTB	Neg	Neg	Bilateral infiltrates, bilateral cavities	None	4	H, R, Z, E
9	15	PTB	Neg	Pos	Bilateral infiltrates	None	2	H, R, Z, E
10	15	PTB	Pos	Pos	Bilateral infiltrates, bilateral cavities, unilateral fibrosis	None	1	H, R, Z, E
11	3	PTB	Pos	Pos	Bilateral infiltrates	Confirmed MDR-TB patient	None	None
12	2	PTB and EPTB (lymphadenitis)	Neg	Neg	Bilateral infiltrates, bilateral cavities	TB patient who died on treatment	2	H, R, Z, E
13	7	PTB and EPTB (miliary)	Pos	Neg	Bilateral miliary pattern	None	1	H, R, Z, E
14	4	PTB	Neg	Neg	Total consolidation, collapsed left lung, unilateral infiltrates	Suspected MDR-TB patient	2	H, R, Z, E
15	2	PTB	Neg	Neg	Bilateral infiltrates	None	3	H, R, Z, E
16	4	PTB	Neg	Neg	Bilateral infiltrates	Confirmed MDR-TB patient	1	H, R, Z, E
17	13	PTB	Neg	No baseline	Unilateral infiltrates and cavity	None	3	H, R, Z, E
18	13	PTB	Pos	Neg	Bilateral infiltrates, bilateral cavities, unilateral fibrosis	None	2	H, R, Z, E, S
19	9	PTB	Pos	Pos	Bilateral cavities, bilateral infiltrates	None	3	H, R, Z
**Drugs to which isolate was resistant** b	**Drugs to which isolate was susceptible** b	**Drugs used during MDR-TB treatment**	**HIV status**	**Baseline CD4 count (cells/mm^3^) (CD4%)**	**ART initiation relative to MDR-TB treatment**	**Outcome or treatment status**		
H, R, S	E	Cm, Mfx, Eto, Cs, PAS	Neg	–	–	Cured after 25.5 months		
No DST results	No DST results	Cm, Mfx, Eto, Cs, PAS	Pos	827	2 months after	Treatment completed after 24 months		
No DST results	No DST results	Cm, Mfx, Eto, Cs, PAS, Z	Pos	815	2 months prior	Died after 18 months		
H, R, Z, S c	E	Cm, Ofx, Eto, Cs, PAS, E, Z	Pos	20	2 weeks after	Cured after 24 months		
None	H, R, E, S	Cm, Ofx, Eto, Cs, PAS, H, R	Pos	46	2 months prior	Cured after 18.5 months		
No DST results	No DST results	Km, Ofx, Eto, Cs, PAS	Pos	486	1 week after	Treatment completed after 22.5 months		
None	H, R, E, S	Cm, Mfx, Eto, Cs, PAS, H, R, E, Z	Neg	–	–	Cured after 20 months		
No DST results	No DST results	Cm, Mfx, Eto, Cs, PAS, Z	Pos	841	1 week prior	Cured after 21 months		
None d	H, R	Cm, Lfx, Pto, Cs, PAS, H, R, Z	Pos	4	2.5 years prior	Cured after 19.5 months		
H, R, E	S	Km, Lfx, Pto, PAS, Z	Neg	–	–	Cured after 22.5 months		
H, R d	None	Cm, Lfx, Pto, Cs, PAS, Z	Pos	780	2 weeks after	Treatment completed after 21 months		
No DST results	No DST results	Cm, Lfx, Pto, Cs, PAS, Z	Neg	–	–	Treatment completed after 18 months		
No DST results	No DST results	Cm, Lfx, Pto, Cs, PAS, Z	Pos	487	1 week after	Treatment completed after 21 months		
No DST results	No DST results	Cm, Lfx, Pto, Cs, PAS, Z	Neg	–	–	Treatment completed after 21 months		
No DST results	No DST results	Cm, Lfx, Pto, Cs, PAS, Z	Pos	452(18%)	1 year prior	Died after 12 months		
No DST results	No DST results	Cm, Lfx, Pto, Cs, PAS, Z	Pos	519	2 months after	Treatment completed after 20 months		
No DST results	No DST results	Km, Lfx, Pto, Cs, PAS, Z	Pos	16	9 months prior	Cured after 22 months		
None e	H, R, E, S	Cm, Lfx, Pto, Cs, PAS, H, R, Z	Pos	59	6 months prior	In 17th month of treatment, smear-negative since second month of treatment		
H, R	E, S	Cm, Lfx, Pto, Cs, PAS, Z	Pos	504	3.5 years prior	In 7th month of treatment, smear-negative since third month and culture-negative since first month		

**Note:** PTB: pulmonary tuberculosis; EPTB: extrapulmonary tuberculosis; Pos: positive; Neg: negative; H: isoniazid; R: rifampicin; E: ethambutol; S: streptomycin; Z: pyrazinamide; Eto: ethionamide; Cfx: ciprofloxacin; Cm: capreomycin; Km: kanamycin; Mfx: moxifloxacin; Ofx: ofloxacin; Lfx: levofloxacin; Cs: cycloserine; Pto: prothionamide; PAS: para-aminosalicylic acid.

a This patient was smear- and culture-positive before commencing first-line TB treatment but culture converted prior to initiating MDR-TB treatment.

b Patients’ isolates were tested for resistance to H, R, E, S using BACTEC MGIT 960 system (Becton-Dickson, Sparks, Maryland, USA), unless otherwise indicated.

c This patient’s isolate was tested for resistance to H, R, E, S, Z using epsylometer test (Etest) on agar plate.

d These patients’ isolates were tested for resistance to H, R using line probe assay (Hain Lifescience GmbH, Nehren, Germany).

e This patient did not have baseline DST results but later had DST results from a positive culture in the tenth month of treatment.

**Table 2:** Baseline characteristics (N = 19).

Characteristic		No. of Patients (%)	Median (Range)
Male		10 (53)	
Age (years)			8 (2–15)
Weight-for-age, z-score a			−1.90 (−3.72 to +0.17)
Height-for-age, z-score a			−2.71 (−6.36 to +2.58)
Malnutrition b		12 (63)	
Stunting c		12 (63)	
HIV co-infection		14 (74)	
Cavitary lesions or bilateral disease on chest radiograph		16 (84)	
Extrapulmonary TB concurrent with pulmonary TB		4 (21)	
Number of previous treatments	None	1 (11)	
	One	7 (37)	
	Two	6 (32)	
	Three or more	5 (26)	
No. of first- and second-line TB drugs exposed to previously			4 (0–6)
Baseline bacteriologic results	Smear-positive and culture-positive	7 (37)	
	Smear-positive and culture-negative	2 (11)	
	Smear-negative and culture-positive	1 (5)	
	Smear-negative and culture-negative	8 (42)	
	Smear-negative and no culture result	1 (5)	
DST results available		9 (47)	
Household contact with laboratory-confirmed MDR-TB patient		3 (16)	
Household contact with suspected MDR-TB patient		6 (32)	
Orphan	Lost one parent	4 (21)	
	Lost two parents	7 (37)	

a Based on the US Centers for Disease Control and Prevention (CDC) clinical growth charts [63].

b Defined as weight-for-age of <5th percentile according to US CDC clinical growth charts.

c Defined as height-for-age of <5th percentile according to US CDC clinical growth charts.

Patients were treated with a median of six antituberculous drugs (range, 5 to 9). Sixteen patients (84%) were hospitalized at treatment initiation for a median of 1.5 months (range, 0.5 to 9) (Table 3). Most of these patients did not have a clinical indication for hospitalization at treatment initiation, but were hospitalized because of unstable household situations and other social problems. Among the 18 patients who completed the intensive phase of treatment, the parenteral agent (kanamycin or capreomycin) was administered for a median of nine months (range, 8 to 12).

**Table 3:** Treatment of MDR-TB and HIV disease.

Characteristic		N (%) or Median (range or IQR)
**MDR-TB Treatment (N = 19)**		
Hospitalization at initiation – n (%)		16 (84)
Length of hospitalization, mo – median (range)		1.5 (0.5–9)
No. of drugs in regimen – median (range)		6 (5–9)
Time on parenteral agent, mo (N = 18) a – median (range)		9 (8–12)
Time to culture conversion, days (N = 7) – median (range)		53 (37–105)
Time to smear conversion, days (N = 8) – median (range)		51.5 (32–93)
Final outcomes (N = 17) – n (%)	Cured	7 (41)
	Completed treatment	8 (47)
	Died	2 (12)
**Antiretroviral Treatment (N = 14)**		
Baseline CD4 cell count (cells/mm^3^) – median (IQR)		486.5 (49.3–714.8)
Increase in CD4 cell count (cells/mm^3^) during MDR-TB treatment – median (IQR)		397 (276.5–622)
Baseline weight (kg) – median (IQR)		16.5 (15–22)
Increase in weight (kg) during MDR-TB treatment – median (IQR)		4 (3–6)
On ART at MDR-TB treatment initiation – n (%)		8 (57)
Started ART after MDR-TB treatment initiation – n (%)		6 (43)
Time to ART initiation after start of MDR-TB regimen (days) (N = 6) – median (range)		13.5 (8–70)
Initial ART regimen concurrent with MDR-TB treatment – n (%)	AZT, 3TC, EFZ	9 (64)
	D4T, 3TC, EFZ	3 (21)
	D4T, 3TC, NVP	1 (7)
	DDI, ABC, RTV/LPV	1 (7)
ART regimen changes during MDR-TB treatment – n (%)	AZT switched to D4T due to anemia	3 (21)
	D4T switched to AZT due to peripheral neuropathy	1 (7)
	DDI, ABC, RTV/LPV switched to AZT, 3TC, EFZ following genotypic resistance testing	1 (7)

**Note:** IQR: interquartile range; AZT: zidovudine, 3TC: lamivudine, D4T: stavudine, EFZ: efavirenz, DDI: didanosine, ABC: abacavir, RTV: ritonavir, LPV: lopinavir.

a Among patients who completed the intensive phase of treatment.

Final outcomes were available for 17 patients: 15 patients (88%) had treatment success (seven patients cured and eight patients completed treatment) and two patients (12%) died (Table 3). Both patients who died (case 3 and 16) were co-infected with HIV. These two children showed significant clinical improvement on second-line TB therapy, including substantial weight gain and an end of TB symptoms. During the eighteenth month of treatment, case 3 arrived at the MDR-TB hospital with severe dyspnea and died the next morning of an unknown cause. Case 16 missed her clinic visit in the twelfth month of treatment, and tracking by the community team revealed that the patient had died at home of an unknown cause. No patients defaulted or were lost to follow-up. The two patients currently on treatment have exhibited favorable responses to MDR-TB therapy.

Eighteen patients (95%) experienced at least one adverse event (Table 4). Most adverse events were mild to moderate in severity and were successfully managed without suspending treatment (Table 4). The most common adverse events were hypothyroidism (79%), hypokalemia (68%), and nausea or vomiting (53%). One patient who had been on ART for more than two years experienced elevated liver enzymes two weeks after initiation of MDR-TB treatment (case 9). MDR-TB treatment was suspended until transaminitis resolved, and after five weeks, the MDR-TB regimen was reintroduced without pyrazinamide. In another patient, cycloserine was permanently discontinued due to vision problems (case 8).

**Table 4:** Frequency of adverse events (N = 19).

Adverse Events	No. of Patients (%)
Any adverse events	18 (95)
Hypothyroidism a	15 (79)
Hypokalemia b	13 (68)
Nausea/vomiting	10 (53)
Rash	8 (42)
Ototoxicity c	6 (32)
Peripheral neuropathy	5 (26)
Headache	4 (21)
Anemia d	3 (16)
Visual problems	2 (12)
Depression/anxiety	2 (12)
Other neurological effects	2 (12)
Hepatotoxicity e	2 (12)
Arthralgia	1 (6)
Nephrotoxicity f	1 (6)
Psychosis	0 (0)
Seizure	0 (0)
Dosage adjustments of one or more TB drugs	2 (12)
Temporary suspension of one or more TB drugs	3 (18)
Permanent discontinuation of one or more TB drugs	2 (12)

a Elevation at least one thyroid-stimulating hormone >10.0 mIU/l (normal range: 0.37–3.50 mIU/l).

b At least one serum potassium value <3.5 mmol/l (normal range: 3.5 to 5.5 mmol/l).

c Based on patient or caregiver self-report, confirmed by audiometry.

d At least one hemoglobin value <11 g/dl (normal range: 11.7 to 16.0 g/dl for females, 13.7 to 17.8 g/dl for males).

e Elevated liver enzymes (normal ranges: ALP 117 to 390 U/l, ALT 0 to 49 U/l, AST 0 to 46 U/l).

f Elevation of at least one serum creatinine value ≥130 µmol/L (normal range: 44 to 115 µmol/L).

Among the 14 patients with HIV co-infection, the median (interquartile range [IQR]) CD4 cell count at the start of MDR-TB treatment was 486.5 (49.3–714.8) cells/mm^3^ (Table 3). Eight patients were already on ART at the start of the MDR-TB regimen, and the remaining six patients were started on ART a median of 13.5 days after the MDR-TB regimen. The median (IQR) increase in CD4 cell count during MDR-TB treatment was 397 (276.5–622) cells/mm^3^, and the median (IQR) weight gain among HIV co-infected patients was 4 (3–6) kg. There were no incidences of immune reconstitution inflammatory syndrome (IRIS) after ART initiation. Three patients were switched from zidovudine to stavudine when they experienced anemia, and one patient was switched from stavudine to zidovudine after the onset of peripheral neuropathy. Another patient who had experienced immunological deterioration prior to MDR-TB treatment was switched from a second-line ART regimen to a first-line regimen when genotypic resistance testing revealed no mutations conferring resistance to first-line antiretroviral drugs.

## Discussion

Our results demonstrate that, even in a setting of high HIV prevalence, it is possible to achieve favorable outcomes among children treated for MDR-TB using early empiric treatment delivered through a comprehensive community-based program. The latter included referral to inpatient care as needed. Almost 90% of the children treated experienced cure or treatment completion, despite the geographically challenging setting and the high rate of baseline malnutrition, severe lung damage, and co-infection with HIV. Excluding the four patients who subsequently had DST results suggesting drug-susceptible TB, the mortality rate among confirmed and suspected MDR-TB cases in this study was 13%. Our outcomes were similar to outcomes among children in MDR-TB treatment programs with largely HIV-negative patients, where treatment success rates have ranged from 80 to 100% and crude mortality rates, from 0 to 6% [25]–[28], [32].

In contrast to the favorable outcomes achieved in low HIV prevalence settings, pediatric case series with a high prevalence of HIV have reported lower rates of treatment success [6], [30]. Both of these case series included only culture-confirmed MDR-TB cases, which may imply more extensive diseases at baseline. One study in South Africa reported final outcomes for 31 children treated for confirmed MDR-TB, of whom six were HIV co-infected and 10 were not tested [6]. Twenty-one children (68%) were cured, four (13%) died, and six (19%) defaulted after at least five months of treatment. However, the study was conducted before ART became available in South Africa. Another study from South Africa reported 12-month outcomes for 13 children with MDR-TB, of whom seven were co-infected with HIV and six received ART [30]. In that report, four children died a median of 2.8 months after submitting sputum for culture and DST, including three co-infected children who were unstable at treatment initiation. Of the remaining nine patients, two defaulted, one patient exhibited radiological deterioration, and six showed improvement by 12 months. Delay in the initiation of appropriate treatment likely played a role in the poor outcomes. Patients initiated MDR-TB treatment a median of 2.5 months after submitting sputum, and three of 13 patients died or defaulted before initiating appropriate treatment.

Two recent studies of DR-TB and HIV co-infected children treated concurrently with ART and second-line TB treatment have reported more favorable outcomes [22], [31]. Four pediatric XDR-TB patients with HIV co-infection were successfully cured with co-treatment in South Africa [22]. Another study in South Africa examined outcomes in 111 children with MDR-TB, including 43 children with HIV co-infection, most of whom initiated ART prior to or during MDR-TB treatment. In that report, 82% of patients achieved favorable outcomes, and five of the 13 deaths occurred before confirmation of MDR-TB and initiation of appropriate treatment [31].

In contrast to previous reports on co-infected children, children in the Lesotho program were initiated on empiric second-line TB treatment immediately after evaluation for suspicion of MDR-TB, without waiting for DST confirmation. Treatment delay is particularly problematic among children because of the difficulty in obtaining sputum and the high likelihood of paucibacillary TB. In a study of 39 children with MDR-TB in South Africa, Schaaf et al. found that treatment delay (the time from when MDR-TB should have been clinically suspected until the initiation of treatment) was a median of two days if the DST of the source case was considered and 246 days if the DST of the source case was not considered [6]. For HIV-infected pediatric patients, guidelines recommend prompt initiation of second-line TB treatment using the index case’s DST results if TB disease develops in a child in close contact with an index case with drug-resistant TB [25], [26], [28], [43].

MDR-TB should be suspected when a child has been in contact with a TB patient who died while on treatment and who was suspected to have had MDR-TB [25], [26]. Six children in our case series had household contact with individuals suspected, but not confirmed, of having MDR-TB. The household source cases had either been initiated on empiric MDR-TB treatment as high-risk MDR-TB suspects or had experienced clinical deterioration of their tuberculosis and died while receiving first-line TB treatment. Given the poor outcomes associated with delayed initiation of treatment, our clinicians initiated empiric second-line therapy in these children.

Efforts to obtain sputum samples for culture and DST confirmation for all pediatric patients were aggressive. Clinicians attempted to collect sputum specimens at monthly clinic visits, and gastric lavage and sputum induction were used for young children. For the diagnosis of extrapulmonary MDR-TB, pleural fluid sample and lymph node aspirates are the only non-pulmonary specimens routinely collected and cultured in Lesotho. Compared with adults, children with TB are more likely to have extrapulmonary TB [7], [44]. However, all patients included in this report had pulmonary involvement. As a result of limited diagnostics for the detection of extrapulmonary TB in this setting, those without pulmonary involvement were likely to have been missed. Moreover, an unusually high proportion of patients had bilateral disease or cavitary lesions, which is not associated with the classic pediatric presentations of TB [44], raising the possibility that significant numbers of children with less severe pulmonary disease may have been undetected.

The five patients with culture-confirmed MDR-TB all experienced cure or treatment completion. Of the other 14 patients, six had household contact with a confirmed MDR-TB patient or high-risk MDR-TB suspect, and the remaining patients had failed to respond to at least one previous course of first-line TB treatment. For several patients who initiated an empiric MDR-TB regimen, subsequent DST results indicated susceptibility to isoniazid and rifampicin. However, all had responded well to MDR-TB treatment, experiencing significant clinical and radiological improvement, after having been unsuccessfully treated with at least one first-line TB regimen, so the clinical team decided to continue them on MDR-TB treatment with the addition of rifampicin and high-dose isoniazid. We did not have data on patients’ adherence to previous courses of first-line TB treatment, and it is possible that non-adherence, despite directly observed therapy, may have contributed to failure of first-line treatment. Additionally, three of the four patients had HIV co-infection, making them susceptible to other potential causes of clinical deterioration during first-line TB treatment [45]. Nevertheless, the clinical team decided it was prudent to continue these four patients on a modified version of the second-line regimen to which they had responded clinically.

Although we were striving to provide rapid and appropriate care to these patients, we note that the majority (58%) of the children in our cohort had already received two or more previous regimens of first-line TB drugs, and all but one of the household contacts had been treated previously with first-line drugs, suggesting that there was a delay in recognizing them as MDR-TB suspects and initiating them on appropriate treatment. Additionally, nearly half of the HIV co-infected patients were not receiving ART at the time of MDR-TB treatment initiation, despite the fact that pulmonary TB is a criteria for initiating ART in the clinical staging of HIV/AIDS (irrespective of CD4 cell count) [46]–[48].

Children with HIV co-infection who were not already on ART were initiated on ART as soon as they were tolerating MDR-TB treatment, as early as one week post initiation. Optimal time to introduce ART following the start of TB treatment is uncertain, as early introduction of ART after initiation of TB treatment is believed to be associated with higher risk of IRIS [49], [50]. However, literature on IRIS in adults suggests that mortality from TB-associated IRIS is rare [51], [52], and earlier initiation of ART has been shown to significantly reduce mortality among severely immunosuppressed adults receiving first-line treatment for drug-susceptible TB [53]. The literature on IRIS in children treated for TB is limited to a small number of case reports and case series [54]–[57]. In a study in South Africa, data from 290 children on ART were analyzed for the clinical presentation and outcome of TB [55]. The authors concluded that the poor outcomes among children who started ART within two months of TB treatment was due to the more advanced HIV disease in this group; the early introduction of ART was rarely (<10% of cases) associated with drug-related adverse events or IRIS. In our program in Lesotho, children responded well to concurrent ART and MDR-TB treatment, with few regimen changes required, no defaults, and no episodes of IRIS observed.

In this cohort, children experienced a higher incidence of adverse events compared to other reports [8], [25], [26], [32]. Nearly 80% of patients experienced hypothyroidism a median of 75 days after MDR-TB treatment initiation. Recent studies from South Africa have also reported high rates of abnormal thyroid function tests (>50%) among children treated for MDR-TB [58], [59]. Although hypothyroidism is a known side effect of ethionamide/prothionamide and PAS, recovery from non-thyroidal illness syndrome (NTIS) could also cause a transient TSH elevation in children with TB or HIV infection. However, TSH was elevated >20 mIU/l in eleven patients, and seven patients experienced prolonged TSH elevation lasting more than three months, suggesting primary hypothyroidism rather than recovery from NTIS.

Patients in this study also experienced a high incidence of hypokalemia (68%), which occurred a median of 39 days after the start of the MDR-TB regimen. In adults, electrolyte disturbances, including hypokalemia, have been attributed to the use of aminoglycosides and capreomycin [60], [61]. Low serum potassium may also result from severe malnutrition or diarrhea and vomiting caused by antituberculous agents, such as PAS, or chronic gastrointestinal infections common among HIV patients. Nearly one-third of patients experienced ototoxicity with a median onset of 265 days after initiating MDR-TB treatment. Since audiometry was performed only to confirm patient or caregiver self-report of hearing loss, this likely represents an underestimate, and routine audiometry should be considered for all children receiving treatment for MDR-TB.

Although ART drugs and second-line TB drugs have potential overlapping or additive toxicities [24], it is not clear that HIV co-infection or concurrent ART could explain the higher incidence of adverse events in the cohort, as adverse events were common among both HIV-infected and HIV-uninfected patients. We suspect that the high degree of malnutrition and poor clinical status at baseline may have played a role. Patients were monitored closely at both the community and clinic level, and adverse events were managed aggressively [37], [41], [60].

In addition to close monitoring of the patients’ clinical status, the CHWs and community team provided close monitoring of the patients’ household situation and psychosocial needs. Many patients had been orphaned by HIV and TB (Table 2), and some had been neglected by their caregivers. The community team helped school-aged children to return to school and assisted families without income to start income generating activities. Providing for these psychosocial needs likely helped to achieve zero defaults and favorable treatment outcomes.

The favorable outcomes reported in this study are particularly notable because they were achieved in a predominately ambulatory setting. Because of the complexity of managing MDR-TB and HIV co-infection, the traditional model of MDR-TB treatment in this region of the world has been hospital-based care, and many children with MDR-TB continue to be hospitalized unnecessarily. The community-based treatment model minimized the risk of nosocomial transmission of MDR-TB, which is particularly important in high HIV prevalence settings like Lesotho. Our findings demonstrate that good MDR-TB outcomes in the context of HIV are achievable using a community-based model. However, successful implementation of community-based care in this geographically challenging setting where many patients live in rural, mountainous areas required several key elements, including strong links between the community team and clinicians, well-trained and supervised CHWs, and reimbursement for travel expenses related to treatment [62].

For children suspected of having MDR-TB disease, particularly those with HIV co-infection, empiric second-line TB therapy should be initiated while awaiting DST results. Patients with HIV co-infection not already receiving ART may benefit from early initiation of ART once the MDR-TB treatment regimen is tolerated. Our community-based treatment delivery model included empiric treatment, as well as close monitoring, frequent routine screening, and timely interventions for adverse events. Treatment outcomes for this strategy are comparable to those reported among children with drug-susceptible TB and those without HIV infection.
